# The Prevalence of Sjögren’s Disease in Dental Clinics in the Netherlands Compared with the Prevalence in a Systematic Literature Review of Studies in Other Countries

**DOI:** 10.3390/jcm13195918

**Published:** 2024-10-03

**Authors:** Floor Maarse, Jitse F. Huisinga, Derk Hendrik Jan Jager, Henk S. Brand

**Affiliations:** 1 Department of Oral and Maxillofacial Surgery/Oral Pathology, Amsterdam UMC, Location Vrije Universiteit Amsterdam, 1081 HV Amsterdam, The Netherlands; f.maarse@amsterdamumc.nl (F.M.); d.jager@amsterdamumc.nl (D.H.J.J.); 2 JFH Dentists, 1901 GG Castricum, The Netherlands; 3 Amsterdam Institute for Infection and Immunity, Inflammatory Diseases, Location Vrije Universiteit Amsterdam, 1081 HV Amsterdam, The Netherlands; 4 Department of Oral Biochemistry, Academic Centre for Dentistry (ACTA), 1081 LA Amsterdam, The Netherlands

**Keywords:** Sjögren’s disease, prevalence, incidence

## Abstract

**Background/Objectives**: Sjögren’s disease (SjD) is an autoimmune disease causing irreversible damage to the exocrine glands but can have symptoms throughout the entire body. The aim of this study is to determine the prevalence of Sjogren’s disease (SjD) in the Netherlands, compare this with the prevalence for other countries in a systematic literature review. **Methods**: In the first part of this study, the prevalence of SjD was determined at two academic dental clinics in the Netherlands by electronically analysing patient records. In the second part of this study, a systematic literature search was performed in PubMed. Studies in the English language reporting prevalence ratios (PRs), incidence ratios (IRs) or sufficient data to calculate these parameters were included. Population-based studies and population surveys aiming to examine an entire geographic region or using a clearly defined sampling procedure were included. Review studies were excluded. Studies that did not report sufficient data or contained no original data were excluded. Included studies were assessed using the Newcastle–Ottawa assessment scale. **Results**: At the dental clinic in Amsterdam, 76 SJD patients were identified among a patient population of 81941, resulting in a prevalence ratio of 93 per 100,000 (0.093%) patients. In Nijmegen, 21 SjD patients were identified in a total patient population of 14,240, resulting in a prevalence ratio of 147 per 100,000 (0.15%). Thirty-one studies were included in the systematic review. They varied in diagnostic criteria for SjD with the American-European Consensus Group (AECG) criteria being the most widely used. The reported prevalence ratio varied from 0.008% to 3.3%. The overall pooled prevalence ratio of SjD using the AECG criteria was 0.031%, while the pooled prevalence of SjD using the EU criteria was 0.029%. The overall pooled incidence ratio was 5.2 (95%CI 4.7 to 5.6) per 100,000 person-years. **Conclusions**: The estimated prevalence ratio of SjD in the Netherlands (0.09% to 0.15%) falls within the worldwide range but is higher than the worldwide pooled prevalence ratio.

## 1. Introduction

Sjögren’s disease (SjD) is a chronic and progressive autoimmune disease causing irreversible damage to the exocrine glands and is associated with the B and T lymphocyte infiltration of the affected glands [1,2]. Although SjD is a systemic disease and can have symptoms throughout the entire body, it mainly affects the lacrimal and salivary glands. The predominant symptoms are dry eyes, hyposalivation and xerostomia [2,3]. Other symptoms are fatigue, joint pain, vaginal dryness and depression [3,4,5,6]. Furthermore, among patients with SjD, the risk of B-cell lymphoma is 15 to 20 times higher than in the general population [6]. SjD can be divided into primary SjD and SjD associated with another rheumatic disease, such as rheumatoid arthritis or systemic lupus erythematosus [3,7]. Due to the decreased saliva secretion, the altered saliva composition [8] and the reduced capability of saliva to buffer, lubricate and perform antimicrobial activities, caries risk in SjD patients is increased [9,10]. SjD patients have an increased risk of root caries and caries on the labial and incisal surfaces of the teeth. Also, a diminished taste [11] and swallowing disorders [12] are frequently reported. Finally, SjD can increase the risk of Candidiasis and the inflammation of the oral mucosa [9]. In the early stages of SjD, salivary flow can be stimulated by the use of lozenges and chewing gums or systematic pharmacotherapies such as pilocarpine or cevimeline [13,14]. Alternatively, sialoendoscopy as a therapeutic procedure [15] and acupuncture [13,16] have also been reported to increase saliva production.

Worldwide, several studies have explored the prevalence of SjD, and large differences have been reported between different parts of the world. This could be related to ethnic and geographical differences [7,17]. Moreover, the use of different classification criteria to diagnose SjD could also explain these differences. Some studies use the International ‘Statistical Classification of Diseases and Related Health Problems’ (ICD) code system, a globally used tool to classify diseases. The ICD system is primarily used for administrative purposes, such as tracking diseases, reimbursement and public health reporting. It does not define specific clinical criteria for the diagnosis of SjD but simply categorizes diseases based on existing definitions. Other frequently used diagnosis criteria are the EU-1993 and EU-1996, Copenhagen and San Diego criteria. For harmonization purposes, the American-European Consensus Group (AECG) proposed in 2002 a new set of criteria, based on previous research by Vitali and co-workers [18]. Finally, in 2016, the ACR/EULAR set of criteria was introduced, which excludes the most common differential diagnoses. It also differs substantially from the previous AECG criteria in that it considers systemic manifestations and introduced a weighted scoring system. [19] The EU-1993 and the San Diego and the Copenhagen criteria could be regard as more symptom-based criteria placing emphasis on patient-reported symptoms of dry eyes and dry mouth. In contrast, the EU-1996, the AECG and the 2016 ACR/EULAR criteria place more focus on objective tests including histopathological examination and antibody testing (anti-Ro/SS-A and anti-La/SS-B), moving away from reliance on subjective symptoms [20]. The use of different classification criteria and the broad nature of ICD coding can create substantial differences in the reported prevalence of Sjögren’s syndrome. More lenient or symptom-based criteria will often lead to higher prevalence estimates, while stringent, objective-based criteria that rely on specific test results will produce lower estimates. This discrepancy is influenced by varying access to diagnostic testing, changing classification standards over time and the administrative nature of the ICD coding system.

The prevalence of SjD in the Netherlands is unknown. Therefore, the aim of our study was to determine the prevalence of Sjögren’s disease at two academic dental clinics in the Netherlands and compare this prevalence with a worldwide systematic literature review of previous studies in other countries.

## 2. Material and Methods

### 2.1. Patient Selection for Prevalence Study

This study was approved on 21 November 2019 by the Internal Ethical Review Board of the Academic Centre for Dentistry Amsterdam (ACTA) under protocol number 201967. To determine the prevalence of SjD within the Academic Centre of Dentistry Amsterdam (ACTA), the electronic health record system Axium (Exan group, Coquitlam, BC, Canada) was automatically searched as previously described [21] for the terms Sjögren, Sjogren, Sjögrens syndrome and Sjogrens syndrome. Only patients who visited the dental clinic in the period from 2010 up to 2019 were included.

In the prevalence study performed at RadboudUMC Nijmegen, patients were identified by an automated search in the electronic health record system Dentium EDU (Netpoint Group, Waalwijk, the Netherlands). The search term ‘Sjogren’ was used to identify patients who visited the dental clinic in the period from 2010 to 2019. Data about the diagnosis of SjD were extracted manually by reviewing the identified health records. In both dental clinics, the prevalence ratio was calculated by dividing the number of SjD patients identified by the total number of patients that enrolled in the clinic during these years.

### 2.2. Systematic Literature Review

#### 2.2.1. Type of Studies

For this research, population-based studies and population surveys aiming to examine an entire geographic region or using a clearly defined sampling procedure were included. Review studies were excluded. Studies were eligible for inclusion if they reported prevalence ratios (PRs), incidence ratios (IRs) or sufficient data to calculate the PRs or IRs. Studies that did not report sufficient data or contained no original data were excluded.

#### 2.2.2. Type of Participants

The selected studies included patients with primary SjD or SjD associated with another rheumatic disease.

#### 2.2.3. Types of Outcome Measures

The prevalence of SjD was the primary variable of interest. The prevalence ratio data included the number of patients with SjD and the size of the study population during the study period. The secondary variables extracted were the author, publication year, country of origin, study period, the patient selection method, patient age and criteria used for the diagnosis of SjD.

#### 2.2.4. Search Strategy, Screening and Selection

The electronic database PubMed was searched using the term *‘Sjogren’s syndrome’* in combination with ‘*prevalence*’ and ‘*epidemiology*’ for studies up to August 2024 using the following search strategy: “Sjogren’s Syndrome” [Mesh] AND (“Prevalence” [Mesh] OR “Epidemiology” [Mesh] OR “Sjogren’s Syndrome” [Mesh] epidemiology OR “Epidemiology” [Mesh] Sjogren’s Syndrome OR “Sjogren’s Syndrome” [Mesh] prevalence OR Epidemiology Sjögren’s Syndrome). Language was restricted to English.

The titles and abstracts of all identified publications were screened by two reviewers. Differences in judgement were resolved through a consensus procedure. If eligible aspects were present in the title or abstract, full-text publications were obtained, fully read and assessed. Publications which fulfilled all selection and inclusion criteria were included for data extraction. The reference lists of the included publications were also manually searched for potentially relevant publications.

#### 2.2.5. Quality Assessment

Included studies were assessed for quality and bias using the Newcastle–Ottawa Quality Assessment Scale (NOS) for case–control and cohort studies [22]. The NOS consists of 8 items, which are divided into 3 domains: selection, comparability and exposure. Assessed items in the ‘’selection’’ domain were the adequacy of the case definition, representativeness of the cases, selection of controls and the definition of controls. In the ‘’comparability’’ domain only the comparability of the cases was assessed, and in the last domain ‘exposure’, the ascertainment of exposure, the same method of case ascertainment and the non-response rate were assessed. One observer (JH) generated the scores of the included publications. When a study fulfilled an item, this was expressed with a “*”. No symbol indicated that the study was not adequate, or it was not clear whether it was adequate. In case–control and cohort studies, a maximum of 8 points could be obtained. In the adjusted version, applied in this review, only a maximum of 5 items could be assessed. In the ‘selection’ domain, the items ‘selection of controls’ and ‘definition of controls’ were not assessed and neither was ‘the ascertainment of exposure’ in the ‘exposure’ domain. These items were scored as NA = not applicable.

#### 2.2.6. Data Extraction

Two review authors (JFH and FM) extracted data independently with the help of data extraction forms, and outcome data were summarized into Review Manager (RevMan 5.3). The details of the study such as the authors, year of publication, prevalence, prevalence ratio, incidence and incidence ratio were extracted for each study and documented in a data sheet. The overall incidence ratio and overall prevalence ratio were calculated as the weighted average of all included studies. Subsequently, the confidence interval for the calculated overall ratios was determined using the sample size calculator for designing clinical research (www.sample-size.net/confidence-interval-proportion/) (accessed on 13 August 2024).

## 3. Results

The ACTA electronic health record system comprised 81,941 patients who enrolled between 2010 and 2020. A total of 76 patients with SjD were identified. The prevalence of SjD at ACTA was 0.093%, and the prevalence ratio was 92.75 per 100,000 persons. At the dental school of RadboudUMC Nijmegen, the electronic health record system comprised 14,240 patients. In this database, 21 patients were labelled as SjD patients. The prevalence was 0.15%, and the prevalence ratio was 147.47 per 100,000 persons.

### 3.1. Literature Study

**Figure 1:**
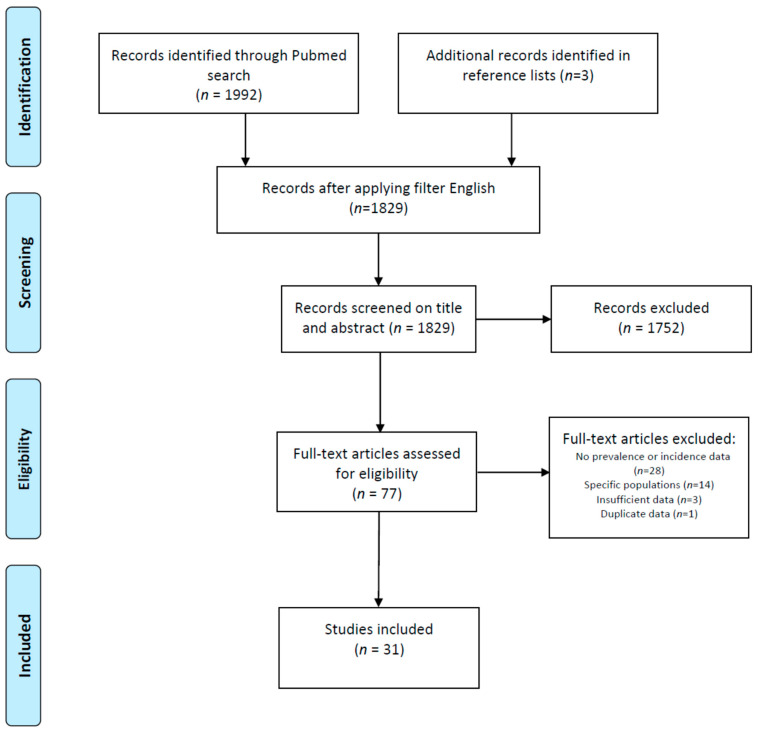
PRISMA flowchart of identification and selection of studies for inclusion.

Initially, 1995 publications were found, and after restriction to publications in the English language, 1829 publications remained. All publications were screened for eligibility based on the title and abstract, after which 77 publications remained. These were screened full text for suitability. Twenty-eight studies were excluded because they did not contain data on the prevalence or incidence of SjD; in three other studies, the data were insufficient to be included, and one study contained data already presented in another publication. Fourteen studies were excluded since they only investigated specific populations. This resulted in 31 included studies. The included studies, of which 21 provided only prevalence ratios, 5 provided only incidence ratios and 5 provided both, were assessed for quality and included for data extraction (Figure 1).

### 3.2. Prevalence Ratio of SjD

Seventeen of the included prevalence studies were conducted in Europe [17,23,24,25,26,27,28,29,30,31,32,33,34,35,36,37,38], five in Asia [39,40,41,42,43], one in the USA [44] and three in South America [45,46,47] (Table 1). Among the included studies were 14 medical record searches, in which the medical history of included patients was screened for SjD and diagnosis criteria, and 11 questionnaires followed by a clinical examination to determine whether patients are indeed suffering from SjD. The remaining studies used an initial telephone survey followed by the screening of medical records when necessary. All studies were published between 1995 and 2024. The American-European Consensus Group (AECG) criteria were the most widely used (12 studies) as diagnostic criteria, followed by 8 studies using ICD criteria, 4 studies using the EU criteria, 2 studies using the 2016 ACR/EULAR criteria and 1 using the San Diego criteria.

One study did not provide information on the number of assessed patients [47]. In the remaining 25 studies, the number of included subjects varied from 332 to 67 million patients with a mean of 4,213,682 patients. In three studies, only female subjects were included [27,28,40]. A total of 8 studies took a sample of patients registered in hospitals or rheumatology clinics, 5 studies took a sample of the national health insurance databases and 14 studies included patients from an entire region or country. Sixteen studies reported the prevalence of patients with primary SjD, three studies reported separate values for pSjD and SjD associated with another rheumatic disease and eight studies reported the prevalence of SjD in general.

The total population of subjects, investigated according to the AECG criteria, comprised 4,158,123 individuals with a total pooled prevalence of 0.031%. The highest prevalence in a study using the AECG diagnostic criteria was 0.72% in Turkey [40]. The lowest prevalence using the AECG was 0.01% in both France [32] and the USA [44]. The total population of individuals screened according to the EU criteria was 118,961 with a pooled prevalence of 0.029%, ranging from 0.22% in Norway [29] to 3.30% in the United Kingdom [33]. The total number of subjects in seven studies with the ICD criteria comprised 94,663,803 individuals with a pooled prevalence of 0.048%, varying from 0.038% in Italy [34] to 0.12% in Colombia 7]. The single study from China that used the San Diego criteria reported a prevalence of 0.30% [43].

### 3.3. Incidence Ratio of SjD

Ten studies reported the incidence ratio of SjD [23,35,41,42,44,48,49,50,51,52] (Table 2). Four studies were performed in Asia [41,42,50,52], four in Europe [23,35,49,51] and two in the USA [44,48]. Of the included studies, three used AECG and one used the EU criteria. Four studies used International Classification of Diseases (ICD) codes, one study used a combination of ICD and ACR-EULAR criteria and one study did not report the diagnosis criteria used.

The overall study population was 118,356,435. The overall pooled incidence rate was 5.2 (95%CI 4.7 to 5.6) per 100,000 person-years. Two studies reported a change in incidence rate over time. Seror et al. [35] reported that the incidence rate declined in the period 2012–2017, while Conrad et al. [51] reported an increase in the years 2017–2019 compared to 2000–2002.

The total population of individuals diagnosed according to the AECG criteria was 25,074,308. The reported incidence rates in the studies using the AECG criteria ranged from 3.5 (95%CI 2.9 to 4.00) per 100,000 person-years in the USA [44] to 6.0 (5.8 to 6.2) per 100,000 person-years in Taiwan [50]. The pooled incidence rate of SjD in subjects evaluated with the AECG criteria was 5.8 per 100,000 person-years (95%CI 5.4 to 6.3).

### 3.4. Quality Assessment

Only 6 of the 31 included studies fulfilled all 5 relevant criteria of the adjusted Newcastle–Ottawa quality assessment (Table 3). Sixteen studies fulfilled four criteria, while 8 studies fulfilled three criteria and 1 study only two criteria. The most frequent missing information concerned the non-response rate, which was lacking in 19 studies, followed by missing information on the comparability of cases on the basis of the design or analysis (missing in 6 studies). The case definition and the representativeness of the cases were both inadequate in four studies. Four other studies did not use the same method of ascertain of cases. 

## 4. Discussion

This study showed considerable variation in the reported prevalence and the incidence of SjD between countries. The observed SjD prevalence in the Netherlands falls within the range of the worldwide prevalence.

The variation in the results might be related to the different classification criteria used for the diagnosis of SjD, as the items of the criteria of the classification systems differ considerably. According to the EU criteria, patients that suffered from pre-existing lymphoma, acquired immune deficiency disease, sarcoidosis or graft-versus-host disease have to be excluded [53]. In the AECG criteria, these exclusion criteria were extended with the use of anticholinergic drugs, previous head and neck radiation treatment and hepatitis C virus (HCV) infection [18]. Furthermore, according to the AECG criteria, patients should not be given anaesthesia while performing Schirmer’s test, and the definition of ‘histopathology’ was slightly stricter than in the EU criteria [18]. Despite the more strict AECG criteria, the total pooled prevalence of SjD in populations investigated with the ACEG criteria was comparable to the pooled prevalence according to the EU criteria (0.031% and 0.029%, respectively). This suggests that more stringent diagnostic criteria do not lead to a lower estimated prevalence in the population.

Three studies [27,28,40] included only female patients. Because SjD mainly affects women (female–male ratio: 9:1) [1], one limitation of a population study on female individuals only is that it will result in the overestimation of the prevalence of SjD in the general population. Moreover, the included studies showed considerable variation in the mean age of the investigated populations, which also could have affected the prevalence of SjD [30]. The peak incidence of SjD is at approximately 50 years of age [6,54], and the median delay between the appearance of the initial symptoms of SjD and the diagnosis is 8.5 years [55]. This will result in a higher prevalence of SjD in studies where the average age of the population is higher than in studies of a younger population. Conversely, studies of younger individuals may lead to the underestimation of the prevalence of SjD.

In addition, the dropout of patients in some studies could also have affected the reported prevalence and incidence ratios. Anagnostopoulos et al., Thomas et al. and Tomšič et al. reported a response rate of 37 to 48% on their questionnaires [24,26,33]. In contrast, Birlik et al. and Haugen et al. reported much higher response rates of 70% and 98%, respectively [30,39]. The exclusion of the dropouts from the results of the study might introduce the possibility of a response bias, as shown in the study by Bowman et al. [27]. Some investigators tried to correct for the non-response, using assumptions from other similar surveys. Thomas et al. [33] assumed that non-responders are likely to be closer to ‘reluctant’ responders, similar to in a study on low back pain [56]. Haugen et al. and Valim et al., who performed a study based on an initial questionnaire followed by a clinical examination, were confronted with another problem [30,46]. Some of the patients refused an appointment for a physical examination by a physician, which was necessary to confirm the diagnosis. Narvaez and co-workers, who used an initial screening by telephone, were confronted during two phases of their studies with people who did not want to cooperate. Firstly, there were many people who did not want to participate in the initial telephone screening. In addition, several people who had indicated a diagnosis of SjD or Sicca during the telephone interview refused to have that confirmed by a rheumatologist. The fact that in a proportion of SjD patients the diagnosis could not be confirmed affects the reliability of the prevalence reported in these studies.

A limitation of the systematic literature study is that only the Pubmed scientific database was searched, and the search was limited to publications in the English language. This may explain why the majority of the included studies originated from Europe and the USA. As ethnic and geographical differences have been reported [7,17], the under-representation of other continents may have affected the estimation of the total pooled prevalence and incidence.

In our study, to determine the prevalence of SjD in the Netherlands, it can be questioned to what extent patients of academic dental clinic centres are representative of the entire Dutch population. Considering that SjD patients more often suffer from oral health problems [57], it is possible that these patients are more frequently referred to an academic dental clinic, resulting in the over-representation of SjD patients in our study population. Furthermore, SjD patients in the Netherlands are less employed than the general Dutch population, and almost half of all SjD patients in the Netherlands receive disability benefits [58]. The cost of treatment at university dental clinics is lower than that of treatment in a regular dental practice in the Netherlands. As a result, we cannot exclude the possibility that, due to a lower socioeconomic status, SjD patients are more likely to be registered in university dental clinics. Also, the available electronic records lacked further information regarding the diagnosis of SjD. It is therefore possible that some patients have reported to suffer from SjD, without this diagnosis being confirmed by a rheumatologist. Altogether, this means that using data from dental university clinics may have resulted in a certain overestimation of the prevalence of SjD in the Netherlands.

## 5. Conclusions

Despite several potential limitations, this review of the relevant scientific literature provided an insight into the worldwide prevalence of SjD. Although the reported prevalence in the individual studies shows a wide variation from 0.008% to 3.30%, the pooled worldwide prevalence of SjD seems to be in the range of 0.03% to 0.05%, depending on the classification system used. The estimate prevalence in the Netherlands differed slightly from the pooled worldwide prevalence at 0.09% to 0.15%, which might be related to the population surveyed. Despite this relatively low prevalence in the general population, it is essential that healthcare providers screen patients for the possible presence of SjD so that patients can receive optimal (oral) healthcare for complications resulting from SjD at an early stage of the disease.

## Figures and Tables

**Table 1:** Overview of included Sjögren’s disease prevalence studies.

Author	Publication Year	Country of Origin	Study Period	Patients Age (yrs)	Diagnostic Criteria Used	Patient Selection Method	Study Population	Number of SjD Patients	Prevalence Rate	Prevalence Ratio per 100,000	Study Design
Alamanos et al. [23]	2006	Greece	1982–2003	Mean 55	AECG	Medical record search	488,435	422 pSS	0.09%	92.8	Population based
Anagnostopoulos et al. [26]	2010	Greece	2007–2008	Mean 51.08	AECG	Questionnaire + clinical examination	1705	4 pSS	0.23%	234.6	Cross-sectional Population survey
Barrio-Cortes et al. [38]	2023	Spain	2015	≥ 18	AECG, 2012 ACR or 2016 ACR/EULAR	Medical record search	6,401,162	4778	0.084%	84	Cross-sectional Population based
Birlik et al. [39]	2009	Turkey	NS	Mean 43.7	AECG	Questionnaire + clinical examination	2835	6 pSS	0.21%	211.6	Population based
Bowman et al. [27]	2004	UK	NS	35–74	AECG	Questionnaire + clinical examination	846 women	2 pSS	0.2%	236.4	Population survey
Cafaro et al. [34]	2024	Italy	2018	Mean 61	ICD 9	Medical record search	4,809,005	1756 pSS	0.038%	38	Population based
Dafni et al. [28]	1997	Greece	1992	NS	EU 1993	Questionnaire + clinical examination	837 women	5 pSS	0.60%	597.3	Population survey
Eaton et al. [29]	2007	Denmark	1977–2001	NS	ICD 10	Medical record search	5,472,032	2615 SS	0.048%	47.8	Population based
Fernández-Ávila et al. [47]	2020	Colombia	2012–2016	NS	ICD 10	Medical record search	NS	58,680 SS	0.12%	120	Population based
Gøransson et al. [17]	2011	Norway	2008	Mean 61.6	AECG	Medical record search	890,255	424 pSS	0.050%	49.7	Population based
Haugen et al. [30]	2008	Norway	1997–1999	40–44 & 71–74	EU 1996	Questionnaire + clinical examination	13,182 & 2864	2 pSS & 9 pSS	0.22% & 1.40%	430.01	Population survey
Izmirly et al. [44]	2019	USA	2007–2009	≥ 18	AECG	Medical record search	1,585,873	260 pSS	0.01%	13.1	Population based
Kabasakal et al. [40]	2006	Turkey	2001–2002	Mean 37.7	AECG	Questionnaire + clinical examination	831 women	6 pSS	0.72%	722	Cross-sectional Population survey
Maldini et al. [31]	2014	France	2008–2011	Mean 57.1	AECG	Medical record search	1,172,482	133 pSS	0.01%	10.2	Population based
Moreno-Quispe et al. [45]	2019	Peru	2016	NS	ICD 10	Medical record search	15,417,345	1301 SS	0.0084%	8.4	Cross-sectional Population survey
Narváez et al. [37]	2020	Spain	2016–2017	NS	AECG	Telephone survey + medical records	4916	16 pSS 4 sSS	0.25% 0.08%	250 81	Cross-sectional Population survey
Sardu et al. [32]	2012	Italy	2009	15–89	ICD 9	Medical record search	25,885	NS SS	0.03%	31	Population based
See at al. [42]	2013	Taiwan	2005–2009	NS	ICD 9	Medical record search	1,000,000	583 SS	0.05%	58.3	Population based
Seror et al. [35]	2024	France	2011–2018	NS	ICD 10	Medical record search	67,000,000	23,848 pSS 14,809 sSS	0.023% 0.016%	23 16	Population based
Stankeviciene et al. [36]	2021	Lithuania	2017–2019	35–74	2016 ACR/EULAR	Medical record search + clinical examination	1405	2 pSS 2 sSS	0.14% 0.14%	140 140	Cross-sectional Population survey
Thomas et al. [33]	1998	UK	NS	18–75	EU 1993	Questionnaire + clinical examination	341	13 SS	3.30%	3300	Population survey
Tomšič et al. [24]	1999	Slovenia	NS	Mean female 52.2 Mean male 56.3	EU 1993	Questionnaire + clinical examination	332	2 pSS	0.60%	602.4	Population survey
Trontzas et al. [25]	2005	Greece	1966–1999	Mean 55	AECG	Questionnaire + clinical examination	8740	13 pSS	0.15%	148.7	Population survey
Valim et al. [46]	2013	Brazil	2000	18-65	AECG	Questionnaire + clinical examination	1205	2 pSS	0.175%	165.9	Cross-sectional Population survey
Yu et al. [41]	2013	Taiwan	2005–2009	NS	ICD 9	Medical record search	963,355	154 SS	0.016%	16.0	Population based
Zhang et al. [43]	1995	China	NS	NS	San Diego	Questionnaire + clinical examination	2066	7 pSS	0.33%	338.82	Population survey

NS = Not Stated.

**Table 2:** Overview of included Sjögren’s disease incidence studies.

Author	Publication Year	Country of Origin	Study Period	Patients Age (yrs)	Diagnostic Criteria Used	Patient Selection	Study Population	Number of SjD Patients	IR (95%CI)/100,000	Study Design
Alamanos et al. [23]	2006	Greece	1982–2003	Mean 55	AECG	Medical record search	488,435	422 pSS	5.3 (4.5–6.1)	Population based
Chen et al. [52]	2022	Taiwan	2002–2012	Mean 42.6	AECG/ACR-EULAR	Medical record search	189,200	1081 pSS	0.63 (0.59–0.67)	Cross-sectional Population survey
Conrad et al. [51]	2023	UK	2000–2019	Mean 54	ICD 10	Medical record search	22,009,375	12,292 SS	6.0-10.7 (NS)	Cross-sectional Population based
Izmirly et al. [44]	2019	USA	2007–2009	Mean 53	AECG	Medical record search	1,585,873	222 pSS	3.5 (2.90–4.1)	Population based
Pillemer et al. [48]	2001	USA	1976–1992	Mean 59	NS	Medical record search	108,145	53 pSS	3.9 (2.8–4.9)	Cross-sectional Population survey
Plešivčnik et al. [49]	2004	Slovenia	2000–2002	Mean 51.3	EU 1996	Medical record search + clinical examination	600,000	71 pSS	3.9 (1.1–10.2)	Population based
See at al. [42]	2013	Taiwan	2005–2009	NS	ICD 9	Medical record search	1,000,000	583 SS	11.8 (10.8–12.7)	Population based
Seror et al. [35]	2024	France	2011–2018	NS	ICD 10	Medical record search	67,000,000	23,848 pSS 14,809 sSS	0.7–4.3 (NS) 0.2–2.0 (NS)	Population based
Weng et al. [50]	2011	Lithuania	2005–2007	Mean 53	AECG	NHI research database	23,000,000	3352 pSS	6.0 (5.8–6.2)	Cross-sectional Population survey
Yu et al. [41]	2013	Taiwan	2000–2008	NS	ICD 9	Medical record search	2,375,407	855 SS	10.6 (9.9–11.4)	Population based

NS = Not Stated.

**Table 3:** Adjursted Newcastle–Ottawa quality assessment.

	SELECTION			COMPARABILITY			EXPOSURE		
	Is the Case Definition Adequate (1)	Representativeness of the Cases (2)	Selection of Controls (3)	Definition of the Controls (4)	Comparability of Cases on the Basis of the Design and Analysis (5)	Ascertainment of Exposure (6)	Same Method of Ascertain for Cases (7)	Non-Respons Rate (8)	Score
Alamanos et al. [23]	*	*	NA	NA	*	NA	*		4
Anagnostopoulos et al. [26]	*	*	NA	NA	*	NA	*	*	5
Barrio-Cortes et al. [38]	*	*	NA	NA	*	NA	*		4
Birlik et al. [39]	*	*	NA	NA	*	NA	*	*	5
Bowman et al. [27]	*		NA	NA	*	NA	*	*	4
Cafaro et al. [34]	*	*	NA	NA		NA	*		3
Chen et al. [52]	*	*	NA	NA	*	NA	*	*	5
Conrad et al. [51]	*	*	NA	NA	*	NA	*	*	5
Dafni et al. [28]	*	*	NA	NA	*	NA	*		4
Eaton et al. [29]	*	*	NA	NA	*	NA	*		4
Fernández-Ávila et al. [47]	*	*	NA	NA	*	NA			3
Gøransson et al. [17]	*	*	NA	NA	*	NA	*	*	5
Haugen et al. [30]	*	*	NA	NA	*	NA	*		4
Izmirly et al. [44]	*	*	NA	NA	*	NA	*		4
Kabasakal et al. [40]	*		NA	NA	*	NA	*	*	4
Maldini et al. [31]	*	*	NA	NA	*	NA	*	*	5
Moreno-Quispe et al. [45]			NA	NA	*	NA	*		2
Narváez et al. [37]	*	*	NA	NA		NA	*		4
Pillemer et al. [48]		*	NA	NA	*	NA	*		3
Plešivčnik et al. [49]	*	*	NA	NA	*	NA	*		4
Sardu et al. [32]	*		NA	NA	*	NA			3
See at al. [42]	*	*	NA	NA	*	NA	*		3
Seror et al. [35]	*	*	NA	NA		NA	*		3
Stankeviciene et al. [36]	*	*	NA	NA		NA		*	3
Thomas et al. [33]	*	*	NA	NA	*	NA		*	4
Tomšič et al. [24]	*	*	NA	NA	*	NA	*		4
Trontzas et al. [25]		*	NA	NA		NA	*	*	4
Valim et al. [46]	*	*	NA	NA	*	NA	*		4
Weng et al. [50]		*	NA	NA	*	NA	*		3
Yu et al. [41]	*	*	NA	NA		NA	*		4
Zhang et al. [43]	*	*	NA	NA	*	NA	*		4

For each criterium: * = adequate and NA = not applicable. Blank means not clear or not adequate.

## Data Availability

The data from the patient survey in the Netherlands are not available due to privacy reasons. The articles on which the systematic review is based are available upon request from the corresponding author.
